# Prognostic Molecular Biomarkers in Breast Cancer Lesions with Non-Mass Enhancement on MR

**DOI:** 10.3390/diagnostics14070747

**Published:** 2024-03-30

**Authors:** Mei-Lin Wang, Yu-Pin Chang, Chen-Hao Wu, Chuan-Han Chen, Mein-Kai Gueng, Yi-Ying Wu, Jyh-Wen Chai

**Affiliations:** 1 Department of Radiology, Taichung Veterans General Hospital, Taichung 407219, Taiwan; wmlovej@gmail.com (M.-L.W.);; 2 Premium Health Examination Center, Tungs’ Taichung MetroHarbor Hospital, Taichung 43503, Taiwan

**Keywords:** magnetic resonance imaging, breast neoplasms, prognosis, retrospective studies

## Abstract

Clustered ring enhancement (CRE) is a new lexicon for non-mass enhancement (NME) of breast MR in the 5th BIRADS, indicating a high suspicion of malignancy. We wonder if the presence of CRE correlates with expression of prognostic molecular biomarkers of breast cancer. A total of 58 breast lesions, which MRI reported with NME, were collected between July 2013 and December 2018. The patterns of enhancement including CRE were reviewed and the pathological results with expression of molecular biomarkers were collected. The association between MRI NME, pathological, and IHC stain findings were investigated under univariate analysis. A total of 58 breast lesions were pathologically proven to have breast cancer, comprising 31 lesions with CRE and 27 lesions without CRE on breast MRI. The expression of the estrogen receptor (ER) (*p* = 0.017) and the progesterone receptor (PR) (*p* = 0.017) was significantly lower in lesions with CRE as compared with those without CRE. The expression of Ki-67 (≥25%) was significantly higher in lesions with CRE (*p* = 0.046). The lesions with CRE had a lower expression ratio of ER (50.71 ± 45.39% vs. 74.26 ± 33.59%, *p* = 0.028). Our study indicated that lesions with CRE may possess different features from those without CRE in molecular expression, bearing a more aggressive behavior.

## 1. Introduction

Clustered ring enhancement (CRE) is a newly added lexicon for non-mass enhancement (NME) in the 5th BIRADS.

Breast cancer is the most frequently diagnosed invasive cancer in the world, and is also the leading cause of cancer death in women in developed and high-income countries [1]. Breast cancer is also the most common female cancer, with an age-standardized incidence rate of 81.0 per 100,000 persons in 2019 in Taiwan [2]. The mortality rate of breast cancer ranked the fourth highest among all cancer types in 2021 in Taiwan. Peak incidence is at 50–59 years old. Although age-standardized incidence rates per 100,000 persons progressively gets higher (as compared with 49.99 in 2006), the age-standardized mortality rate per 100,000 persons did not significantly increase (13.8 in 2021 vs. 10.41 in 2006) [2,3]. Good prognosis may result from the improvement of screening policy, diagnostic tools, and treatment planning. Female mammographic screening as a public health policy started in 2002, and some women have decided to perform self-paid breast MRI.

Medical advances have transformed the previously surgical-only disease into a multidisciplinary approach for breast cancer. Precise diagnosis depends on the pathological results of an invasive procedure or operative surgery. In some circumstances, reoperation is needed, but the patients may hesitate. If a noninvasive examination can provide more information in treatment planning at the pre-operative stage, individualized surgical and adjuvant therapy may be set up.

Breast MRI is a useful modality for breast cancer detection, lesion range definition, and staging in newly diagnosed patients with high sensitivity and specificity. Gadolinium-enhanced dynamic series are also helpful due to neovascularization in the lesions [4]. In the 4th edition of BIRADS, NME was first described as a lexicon group in breast MR, as one kind of the abnormal enhancing breast lesions other than a mass.

According to the 5th BIRADS, clustered ring NME is defined as “thin rings of enhancement clustered together around the ducts”. Increasing evidence in recent years indicates that clustered ring enhancement correlates with malignant tendency [5,6].

There are several credible molecular biomarkers for predicting disease outcomes and systemic treatment effects, including expression of the estrogen receptor (ER), progesterone receptor (PR), human epidermal growth factor receptor 2 (HER2), and Ki-67. The treatment planning and the disease prognosis differ greatly depending on the different types of immunohistochemical (IHC) staining results [7]. As Moffa et al. [8] proposed that rim enhancement and intralesional necrosis could be positive predictors for triple-negative breast cancer, we wonder if CRE NME could be another predictor of prognostic molecular biomarker expression of breast cancer.

The purpose of this study was to retrospectively investigate the relationship between CRE NME and prognostic molecular biomarkers of breast cancer.

## 2. Material and Methods

### 2.1. Study Population

**Figure 1:**
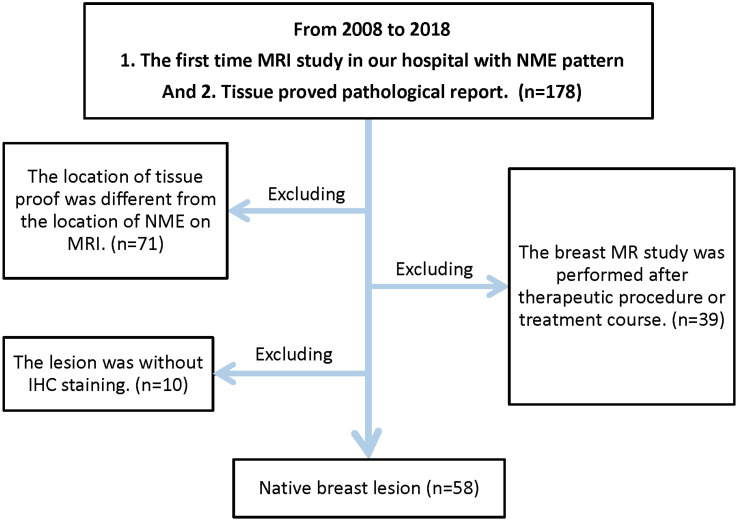
The flowchart of patient enrollment.

A retrospective analysis of the MRI database from Taichung Veterans General Hospital, a tertiary academic hospital, was conducted from July 2013 to December 2018. It was approved by our internal review board. A total of 56 female patients were enrolled. Two of the patients had bilateral lesions at initial interpretation, and thus, 58 lesions of breast carcinoma were recorded and evaluated. The flowchart of patient enrollment is shown in Figure 1.

The criteria for selecting the patients and the lesions were as follows: (a) We searched our breast MRI database for cases with the keywords “non-mass-like enhancement” or “non-mass enhancement” at the time of initial interpretation. (b) Patient who was pathologically diagnosed with breast cancer or carcinoma in situ and had at least one immunohistochemistry staining of four selective markers, including estrogen receptor (ER), progesterone receptor (PR), human epidermal growth factor receptor 2 (HER2), or Ki-67. (c) Patients who received treatment, such as operation, chemotherapy, radiotherapy, or excisional biopsy, were excluded. (d) Patients with pathological sampling collected after chemotherapy or hormone treatment were excluded.

### 2.2. MRI Protocol

Breast MRI was performed with the patient in a prone position using a 1.5-T commercially available system (Magnetom Aera, Siemens Healthcare, Erlangen, Germany) with a dedicated surface breast coil (16 channel and 18 channel).

Our imaging protocols included a localizing sequence followed by an axial fat-suppressed (SPAIR) T2-weighted fast spin-echo, and an axial spin-echo T1-weighted non-fat-suppressed sequence, DWI/ADC before contrast material administration.

Dynamic T1-weighted fat-suppressed 3D gradient-echo sequences (flip angle 12.0 degree; field of view, 320 × 320 mm^2^) were then performed before, and four times after (at approximately 99-s intervals), a bolus intravenous injection of gadobutrol (Gadovist^®^; Bayer Healthcare, Berlin, Germany) (1.0 mmol/mL injection) (2 mL/s) at a dose of 0.1 mmol/kg body weight in the axial plane. Other parameters were field-of-view 320 mm, section thickness 1.5 mm, and interslice gaps 20%.

The same imaging protocol was used for both screening and diagnostic indications.

### 2.3. Image Interpretation

All images of the total 56 MRI examinations enrolled were reviewed without information from pathological reports. The patterns of non-mass enhancement on breast MRI were reviewed according to the 5th BIRADS lexicon. The post-contrast imaging on the axial, sagittal, and coronal plane were evaluated. NME distribution (focal, linear, segmental, regional, multiple regional, or diffuse) and internal enhancement patterns (homogeneous, heterogeneous, clumped, or CRE) were recorded.

The description statistics were conducted for NME patterns and their corresponding pathology. If the enhancing area had more than one feature, we tended to choose the lexicon of the largest part.

For two NME lesions located on different sides of the breasts of one patient, the lesions on different sides were separately interpreted and were regarded as two cases. For the multiple NME lesions on one side of the breast, they were regarded as one single case, and we chose the largest NME lesion to interpret.

### 2.4. Pathological Results

There were cases initially diagnosed by core needle biopsy and cases by both core needle biopsy and surgery. All tissue samples were formalin-fixed and paraffin-embedded sections. Histological types were defined according to the World Health Organization classification [9].

The analysis of the expression of molecular biomarkers was performed by IHC staining. IHC staining was performed separately by using monoclonal primary antibodies (Ventana Medical Systems, Tucson, AZ, USA) for the estrogen receptor (ER) (SP1), progesterone receptor (PR) (1E2), human epidermal growth factor receptor 2 (HER2) (4B5), and Ki-67.

When the result of HER2 was doubtful, gene amplification was verified by in situ hybridization techniques. Detection procedures followed the manufacturer’s instructions for a fluorescence in situ hybridization (FISH) kit for the detection of HER2 amplification (Ventana INFORM HER2 Dual ISH DNA probe cocktail assay).

We searched for and recorded the pathological diagnosis and the IHC staining results of ER, PR, Ki-67 percentage, and FISH of HER2 through reports for every patient with NME on their MRI. ER, PR, and Ki-67 were recorded as percentage positive tumor nuclei in the sample on testing in the presence of expected reactivity of internal (normal epithelial elements) and external controls. The IHC staining result of HER2 is according to the American Society of Clinical Oncology (ASCO)/College of American Pathologists (CAP) guideline. HER2 positivity was considered as score 3+ by IHC or FISH positive, whereas cases with score 0 to 1+ or 2+ without FISH positive were regarded as negative.

### 2.5. Statistical Analysis

Statistical analyses were carried out using SPSS software, version 19.0, (SPSS, Inc., Chicago, IL, USA).

We performed univariate analysis to evaluate the association between MRI NME imaging features and pathological and IHC stain findings. The association between variables was analyzed using the Pearson chi-square or Fisher exact tests for categorical data and the Student’s *t*-test for continuous data. Variables were found to be significant on univariate analysis (*p* value < 0.05).

## 3. Results

### 3.1. Demographics of the Study Population and MRI Patterns

We analyzed 58 malignant breast lesions as the study population, and its demographics are summarized in Table 1. The ratio of CRE and non-CRE of our study population was 31 cases (53.4%) and 27 cases (46.6%), with the latter composed of 17 cases (29.3%) of clump enhancement, 8 cases (13.8%) of heterogeneous enhancement, and 2 cases (3.4%) of homogeneous enhancement. CRE was the most common enhancing pattern of our study.

**Figure 2:**
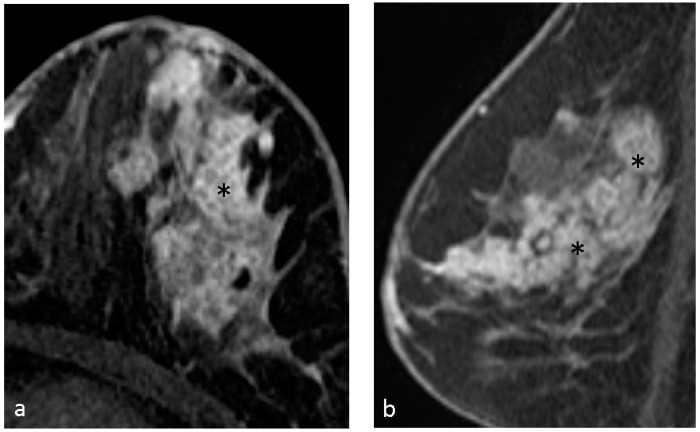
A 55-year-old woman with left breast palpable lesion for several years and with nipple serous discharge for 2–3 weeks. Axial (**a**) and sagittal (**b**) T1-weighted contrast-enhanced MR imaging shows clustered ring NME lesion (*) with segmental distribution. Pathological results of left breast revealed ductal carcinoma in situ.

**Figure 3:**
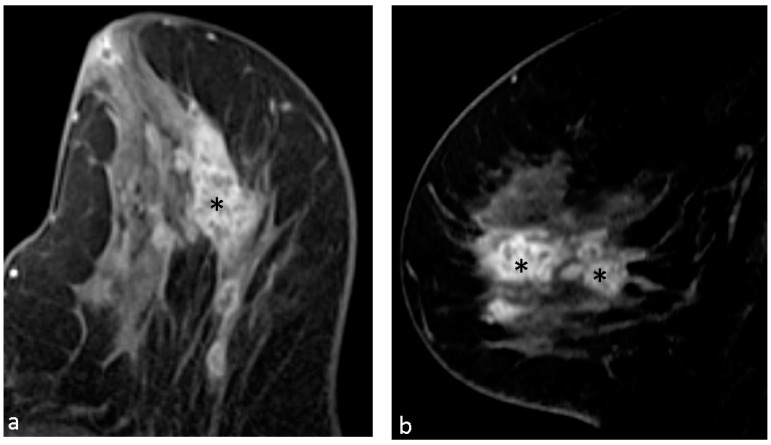
A 77-year-old woman with left breast mastalgia for one week. Axial (**a**) and sagittal (**b**) T1-weighted contrast-enhanced MR imaging shows clustered ring NME lesion (*) with segmental distribution. Pathological results of left breast revealed infiltrating duct carcinoma and ductal carcinoma in situ.

**Figure 4:**
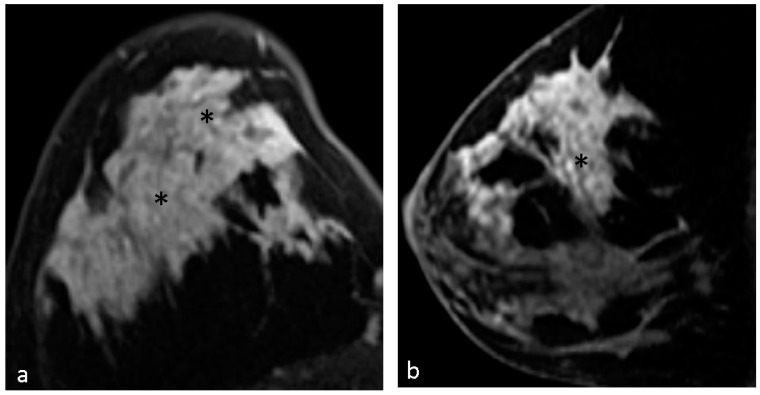
A 36-year-old woman with right breast mass for one month and red nipple discharge. Axial (**a**) and sagittal (**b**) T1-weighted contrast-enhanced MR imaging shows clustered ring NME lesion (*) in right breast with multifocal distribution. Pathological results of right breast revealed infiltrating duct carcinoma and ductal carcinoma in situ.

The distribution of the breast lesions was categorized as focal 6 (10.3%), linear 2 (3.4%), segmental 19 (32.8%), regional 10 (17.2%), multiple regions 17 (29.3%), and diffuse 4 (6.9%). Segmental and multiple regions were the most common enhancing distribution in our study. Figure 2, Figure 3 and Figure 4 demonstrate typical cases with CRE NME with segmental or multifocal distribution.

### 3.2. Histological Types

A total of 58 breast lesions were pathologically diagnosed breast malignancy, including 19 (32.8%) as invasive ductal carcinoma (IDC) only, 23 (39.7%) as ductal carcinoma in situ (DCIS) only, 10 (17.2%) as DCIS with IDC, 4 (6.9%) as invasive lobular carcinoma or lobular carcinoma in situ, and 2 (3.4%) as DCIS (with or without IDC) with lobular cancerization. Pure DCIS was the most common histological type in our study.

The ratio of different NME MRI patterns, distributions, and pathological diagnoses are listed in Table 2.

### 3.3. Age and Demographics

The mean age of our cases was 48.6 years (29–75 years). For the mean age at initial interpretation, the groups of CRE (47.39 ± 10.58 years) and non-CRE (49.96 ± 10.63 years) showed no significant difference (*p* = 0.36).

#### 3.3.1. IHC and CRE

Table 3 and Table 4 show the categorical and quantitative comparison between clustered ring enhancement and prognostic molecular biomarkers in breast cancer.

The statistical results comparing the clustered ring enhancement and the categorical data of prognostic molecular biomarkers of breast cancer are summarized in Table 3. On the other hand, the statistical results comparing the clustered ring enhancement and continuous data of prognostic molecular biomarkers of breast cancer are summarized in Table 4.

All of the 58 breast lesions had ER and PR staining.

ER showed an expression in 74.14% (43/58) breast lesions among the cases. PR showed an expression in 65.52% (38/58) breast lesions among the cases. Expression of ER and PR was significantly lower (*p* = 0.017, *p* = 0.017) in lesions with CRE compared with those without CRE. The breast lesions with CRE pattern tended to exhibit a lower expression ratio of ER than those without CRE pattern (50.71 ± 45.39% vs. 74.26 ± 33.59%, *p* = 0.028). The ratio of PR expression in breast lesions with CRE pattern and without CRE pattern exhibited no significant difference (26.61 ± 34.65% vs. 45.74 ± 41.87%, *p* = 0.066).

#### 3.3.2. Association between Ki-67 and CRE

We used Ki-67 level ≥ 25% as the cut-off point of the Ki-67 proliferative index since better prognostic power was noted according to the previous study [10]. IHC staining of Ki-67 was performed on 38 breast lesions, of which 17 had a high Ki-67 proliferative index. The expression of Ki-67 (≧25%) in lesions with CRE was significantly higher (*p* = 0.046) than those without CRE. The Ki-67 proliferation index of breast lesions with CRE pattern and without CRE pattern exhibited no significant difference (33.90 ± 22.41% vs. 24.33 ± 23.27%, *p* = 0.205)

#### 3.3.3. Association between HER2 and CRE

HER2 IHC staining was performed on 39 breast lesions. All of the lesions with results of 2+ or 3+ received the FISH test. A total of nine lesions had HER2 positive results. There was no significant difference in HER2 overexpression between lesions with CRE and those without CRE.

## 4. Discussion

### 4.1. Malignant Features of NME

Several studies reported that clustered ring enhancement and segmental distribution have the strongest relation with malignancy as compared with other NME features in the 5th BIRADS [5,11,12,13]. Clustered ring enhancement is considered a higher risk of malignancy and more aggressive behavior as compared with clump enhancement, heterogeneous enhancement, and homogeneous enhancement [6].

Tozaki et al. [5] first provided the concept of clustered ring enhancement of ductal carcinoma in situ in MR images. The study showed that segmental distribution and clustered ring enhancement have the highest positive predictive values (PPV) for malignancy, 100% and 96%, respectively. The specificity of clustered ring enhancement for malignant lesions is 63% [5]. Sakamoto et al. proposed that among all the imaging parameters of NME lesions on breast MR, clustered ring enhancement (67%) (*p* = 0.004), branching-ductal pattern (38%) (*p* = 0.003), and clumped architecture (20%) possessed the highest predictive value for cancer prediction [14]. Other studies conducted by Yang, Lunkiewicz, and Chikarmane [11,12,13], respectively, all demonstrated that CRE pattern and segmental distribution are significant indicators distinguishing malignant breast lesions. In addition to the clumped, CRE internal enhancing pattern, Machida et al. [15] proposed another two malignant internal enhancing patterns—branching and hypointense area—whereas CRE and hypointense area were integrated into one collective descriptor called the “heterogenous structures”.

Another study conducted by Liu and Ba et al. found that the distribution (odds ratio (OR) = 8.70), internal enhancement pattern (OR = 6.29), ADC value (OR = 4.56), and vascular sign (OR = 2.84) of the lesions were independent predictors of malignant lesions [16]. They also performed a multimodal scoring analysis using these four predictors, and the analysis revealed diagnostic specificity of 87.01% and sensitivity of 82.22% under the optimal cut-off value of 5. For the independent predictor of internal enhancement pattern, in contrast to our study, they found more malignant lesions demonstrated clumped enhancing pattern (46/77) rather than clustered ring enhancing pattern (2/77), probably due to the different determination of the clustered ring enhancing pattern in their study from others.

### 4.2. Hypothesis of Formation of CRE

Two hypotheses of clustered ring enhancement are contrast media accumulation in the periductal stroma or ductal wall [15], or intraductal wash-in and washout appearance with the scan time at the washout phase [11].

### 4.3. Pathological Diagnosis in NME

The most common pathological finding of NME is pure DCIS in our study. The result is compatible with previous studies [15]. On the other hand, most DCIS, about 60–81% of cases, was interpreted as NME on the MRI [17]. NME can also be seen in invasive breast cancer, benign lesions, and even normal breast tissue [18].

According to the study conducted by Bartels, Fadare et al. [19], NME identified on breast MR carried a significant risk (32%) of atypia and malignancy, which warranted the necessity of biopsy evaluation. Among these atypia or malignant lesions, DCIS was the most commonly identified malignancy (69.2%), whereas the remaining 30.8% were invasive carcinomas.

### 4.4. Pathological Diagnosis in CRE Lesions

Uematsu et al. [20] reported that 77% of CRE lesions were malignant. Of the malignant lesions, 55% were DCIS and 45% were invasive cancers. In the study by Machida [15], 54% of CRE lesions were invasive cancers and 46% of CRE lesions were carcinoma in situ. Their study revealed that both CRE and hypointense areas were significantly associated with invasion. Another study reviewed by two radiologists found that CRE was significantly associated with invasive cancer (*p* = 0.001 and *p* < 0.001, respectively), but there was an absence of necrosis (both *p* < 0.001). Interestingly, they mentioned that clumped enhancement was associated with DCIS (*p* = 0.025 and 0.001, respectively), but also with the presence of necrosis (*p* = 0.003 and 0.001, respectively) [21].

### 4.5. CRE and Biomarkers of ER, PR

ER and PR are two IHC staining markers frequently seen in breast cancer (75–80%) [7]. Several studies showed that the higher percentage of ER and PR staining, the less aggressive behaviors of the cancer [4,22]. They are also prognostic markers of response to treatment [4,22,23]. We found that clustered ring enhancement had a significantly negative correlation with the two hormone biomarkers.

Breast cancers can be divided into luminal types (including luminal A and B) and non-luminal types (HER2-enriches and triple-negative) [7]. The relationship between non-luminal breast cancer (with negative ER and PR staining) and clustered ring enhancement on MR images should be further investigated.

Furthermore, the percentage of ER staining has a significant difference (*p* = 0.028) between groups with and without CRE (50.71 ± 45.39 vs. 74.26 ± 33.59). A lower mean value of the percentage of PR staining is noted in the group with CRE cases with clustered ring enhancement NME, but without significance (CRE: 26.61 ± 34.65 vs. non-CRE: 45.74 ± 41.87, *p* = 0.066).

Due to the clinically aggressive behavior (high metastatic potential, high risk of local recurrence) and distinctive demographics of triple-negative breast cancer, its imaging appearance is of high importance. On breast MR, most cases appear as an oval or round mass with a circumscribed margin, thick/irregular rim enhancement, and high signal intensity on T2-weighted images [24,25,26,27,28]. NME is not a typical imaging appearance of triple-negative breast cancer, but it was reported that 16.0–22.7% cases of triple-negative breast cancer cases demonstrated NME on breast MR [28,29]. It is noteworthy that peritumoral edema, which could be found in 52% of triple-negative breast cancer cases [28], can sometimes be misdiagnosed as NME [30].

### 4.6. CRE and Biomarkers of Ki67

Ki-67 is proven to be a proliferative marker and strong prognostic indicator for overall survival and disease-free survival [22,31]. A previous study by Lee et al. [21] has shown that high Ki-67 expression may correlate with CRE pattern (*p* = 0.048 and 0.003, reviewed by two radiologists), but there was no correlation between HER2 overexpression and enhancing pattern. The results are similar with our findings.

Thus, CRE pattern should be considered as a more aggressive and invasive feature of breast lesion.

## 5. Limitation

First, this was a retrospective, single-institution study, and the case capacity was relatively small. Second, there was probably selection bias in our study. Only cases with lesions that were interpreted as NMEs by a single radiologist at the time of initial interpretation were included in our study. On the other hand, for lesions with cluster ring NMEs but without subsequent surgery, if the lesion could not be observed on a sonography or mammography, their pathology could not be obtained due to the lack of an MR-guided biopsy in our institution.

## 6. Conclusions

Our results indicated that lesions with CRE on breast MRI are different from those without CRE in molecular expression. They bear a more aggressive biological behavior.

## Figures and Tables

**Table 1:** Demographics of the study groups.

Patient No.	56 *
Mean age	48.6 years (29–75 years)
Health exam	1
Clinics	55
Biopsy	35
Operation	23
NME No.	58 *
Immunohistochemical staining No.	
ER	58
PR	58
Ki67	38
HER2	39

* Two patients have bilateral malignant NMEs. NME = Non-mass enhancement, ER = estrogen receptor, PR = progesterone receptor, HER2 = human epidermal growth factor receptor 2.

**Table 2:** Ratio of the 58 malignant breast lesions with different NME MRI patterns, distributions, and pathological diagnoses.

NME Pattern	No. (%)
Clustered ring enhancement	31 (53.4%)
Clump enhancement	17 (29.3%)
Heterogeneous enhancement	8 (13.8%)
Homogeneous enhancement	2 (3.4%)
NME Distribution	
Focal	6 (10.3%)
Linear	2 (3.4%)
Segmental	19 (32.8%)
Regional	10 (17.2%)
Multiple regions	17 (29.3%)
Diffuse	4 (6.9%)
Pathological Diagnosis	
IDC	19 (32.8%)
DCIS	23 (39.7%)
both DCIS with IDC	10 (17.2%)
LCIS with or without ILC	4 (6.9%)
DCIS (with or without IDC) with lobular cancerization	2 (3.4%)

NME = Non-mass enhancement, IDC = invasive ductal carcinoma, DCIS = ductal carcinoma in situ, ILC = invasive lobular carcinoma, LCIS = lobular carcinoma in situ.

**Table 3:** Relationship between CRE and prognostic molecular biomarkers in breast cancer.

Parameters	With CRE	Without CRE	*p*-Value
ER			
Positive	19 (32.8%)	24 (41.4%)	
Negative	12 (20.7%)	3 (5.2%)	0.017 *
PR			
Positive	16 (27.6%)	22 (37.9%)	
Negative	15 (25.9%)	5 (8.6%)	0.017 *
Ki-67			
≥25%	12 (31.6%)	5 (13.2%)	
<25%	8 (21.1%)	13 (34.2%)	0.046 *
HER2			
Positive	5 (12.8%)	4 (10.3%)	
Negative	16 (41.0%)	14 (35.9%)	0.907

Analyzed using the Pearson chi-square or Fisher exact tests. CRE = clustered ring enhancement, ER = estrogen receptor, PR = progesterone receptor, HER2 = human epidermal growth factor receptor 2. * *p* < 0.05 was considered significant.

**Table 4:** Quantitative comparison for prognostic molecular biomarkers and CRE.

Parameters	NME Features	No./Total	Mean ± SD(%)	*p*-Value
ER	With CRE	31/58	50.71 ± 45.39	
	Without CRE	27/58	74.26 ± 33.59	0.028 *
PR	With CRE	31/58	26.61 ± 34.65	
	Without CRE	27/58	45.74 ± 41.87	0.066
Ki67	With CRE	20/38	33.90 ± 22.41	
	Without CRE	18/38	24.33 ± 23.27	0.205

Analyzed using Student’s *t*-test. CRE = clustered ring enhancement, NME = Non-mass enhancement, ER = estrogen receptor, PR = progesterone receptor. * *p* < 0.05 was considered significant.

## Data Availability

All datasets used in this study are available upon request from reviewers and editors.

## Abbreviations

NME	Non-mass enhancement
CRE	Clustered ring enhancement
ER	Estrogen receptor
PR	Progesterone receptor
HER2	Human epidermal growth factor receptor 2
IHC staining	Immunohistochemical staining
